# Densification in transparent SiO_2_ glasses prepared by spark plasma sintering

**DOI:** 10.1038/s41598-022-18892-4

**Published:** 2022-08-30

**Authors:** Hirokazu Masai, Hiromi Kimura, Naoyuki Kitamura, Yuka Ikemoto, Shinji Kohara, Atsunobu Masuno, Yasuhiro Fujii, Takamichi Miyazaki, Takayuki Yanagida

**Affiliations:** 1 Department of Materials and Chemistry, National Institute of Advanced Industrial Science and Technology, 1-8-31 Midorigaoka, Ikeda, Osaka 563-8577 Japan; 2 Graduate School of Materials Science, Nara Institute of Science and Technology, 8916-5, Takayama-cho, Ikoma, Nara, 630-0192 Japan; 3 Japan Synchrotron Radiation Research Institute (JASRI/SPring-8), 1-1-1, Kouto, Sayo-cho, Sayo-gun, Hyogo 679-5198 Japan; 4 Research Center for Advanced Measurement and Characterization, National Institute for Materials Science, 1-2-1, Sengen, Tsukuba, Ibaraki 305-0047 Japan; 5 Graduate School of Science and Technology, Hirosaki University, 3 Bunkyo-cho, Hirosaki, Aomori 036–8561 Japan; 6 Department of Physical Sciences, Ritsumeikan University, 1-1-1 Noji-higashi, Kusatsu, Shiga 525-8577 Japan; 7 Technical Division, Graduate School of Engineering, Tohoku University, 6-6-11, Aoba, Sendai, 980-8579 Japan; 8 National Institute of Advanced Industrial Science and Technology, 1-1-1 Umezono, Tsukuba, Ibaraki 305-8568 Japan

**Keywords:** Glasses, Glasses, Characterization and analytical techniques, Structure of solids and liquids

## Abstract

Recently, spark plasma sintering (SPS) has become an attractive method for the preparation of solid-state ceramics. As SPS is a pressure-assisted low-temperature process, it is important to examine the effects of temperature and pressure on the structural properties of the prepared samples. In the present study, we examined the correlation between the preparation conditions and the physical and structural properties of SiO_2_ glasses prepared by SPS. Compared with the conventional SiO_2_ glass, the SPS-SiO_2_ glasses exhibit a higher density and elastic modulus, but a lower-height first sharp diffraction peak of the X-ray total structure factor. Micro-Raman and micro-IR spectra suggest the formation of heterogeneous regions at the interface between the SiO_2_ powders and graphite die. Considering the defect formation observed in optical absorption spectra, reduction reaction mainly affects the densification of SPS-SiO_2_ glass. Hence, the reaction at the interface is important for tailoring the structure and physical properties of solid-state materials prepared by the SPS technique.

## Introduction

Ceramics have attracted attention from the viewpoint of functional materials with excellent thermal stabilities and chemical durabilities. Considering the Sustainable Development Goals (SDGs), the fabrication of functional materials is not only important scientifically but also environmentally. Because transparent ceramics are used for various optical applications, the available fabrication methods and materials have been investigated worldwide. The synthesis of conventional ceramics require energy, and an energy-less fabrication process is an important aspect of SDGs. One preparation method for obtaining transparent materials is spark plasma sintering (SPS)^1–10^. In contrast to conventional methods for ceramic synthesis, the SPS process enables sintering at a lower temperature and a shorter time by utilising electrical energy and the high energy of discharge plasma^1^. Although the size of the obtained solid is limited, sintering at a high pressure and low temperature is attractive for the preparation of novel functional materials, such as phosphors^7–10^.

It has been reported that the properties of solid materials obtained by SPS are different from those of conventional materials or materials obtained by conventional sintering. Because the SPS technique has been developed for fabrication of materials, functionality seems to be the top priority for researchers. Detailed studies of physical and structural properties are important not only for the analysis of materials but also for the improvement of the SPS technique. Nevertheless, microscopic structural analysis by spectroscopy is considered secondary to the examination of functionality.

SiO_2_ glass has been used as a fundamental optical material with high durability and chemical stability in optical fibres and substrates. Because of the high temperature involved in the fabrication, several attempts have been made to fabricate SiO_2_ glass at low temperatures; these include liquid-phase methods (such as sol–gel) and SPS. SiO_2_ glass prepared by SPS was first reported in the 1990s. The luminescence of SiO_2_ glass containing activators prepared by sintering has been also reported^7–10^. Because SiO_2_ glasses prepared by different methods exhibit different properties for different purposes and applications, it is very important to investigate the relationship between the structure and the physical properties of the prepared SiO_2_ glass. However, a detailed study of the structure of SPS-SiO_2_ glass is lacking. In this study, we performed a structural analysis of SiO_2_ glass prepared by the SPS method and compared its characteristics with those of conventional SiO_2_ glass. In addition, space-selective microscopic analysis was used to determine the spatial heterogeneity of SPS-SiO_2_ glass.

## Results and discussion

### Optical and physical properties

The obtained SPS-SiO_2_ glasses were transparent to the naked eye. In the previous report, SiO_2_ glasses were prepared in the temperature range of 727–1427 °C at 100 MPa pressure, but transparent bulk material was obtained with the sintering temperature above 1250 °C^6^. Since transparent SPS-SiO_2_ glass is the target of the study, four preparation conditions were used: 6 MPa, 1300 °C; 6 MPa, 1400 °C; 70 MPa, 1300 °C; and 70 MPa, 1400 °C. The physical properties of the prepared glasses are listed in Table 1. All the SPS-SiO_2_ glasses exhibit higher densities than that of conventional SiO_2_ glass. In addition, the dense SPS-SiO_2_ glasses possess a higher *G*_0_, *E*_0_, and *K*_0_ than those of conventional SiO_2_ glass. Despite the same preparation conditions, the densities of the SPS-SiO_2_ glasses were changed depending on the weight of starting chemicals. The lighter the starting material, the heavier the density. Notably, the dependence of the preparation conditions (temperature and pressure) on the elastic properties is obscure. Therefore, it is expected that not only the temperature, pressure, and heating program but also the volume of the starting material affects the nature of the obtained samples. Considering the mechanism of the SPS method, the distance between the graphite punches might affect the sintering efficiency and the resulting properties^14–16^.

**Table 1:** Physical properties of the SPS-SiO_2_ glasses compared with those of conventional glass.

ID	Density (g cm^−3^)	Longitudinal sound velocity *V*_L_ (km/s) (± 0.01)	Transverse sound velocity *V*_T_ (km/s) (± 0.01)	Shear Modulus *G*_0_ (GPa) (± 0.1)	Poisson Ratio ν (± 0.001)	Young Modulus *E*_0_ (GPa) (± 0.1)	Bulk Modulus *K*_0_ (GPa) (± 0.1)
Conventional SiO_2_ glass	2.196 (± 0.001)	5.96	3.79	31.6	0.160	73.3	35.9
1300 °C 70 MPa	2.221 (± 0.001)	6.04	3.80	32.0	0.171	75.0	38.0
1300 °C 6 MPa	2.220 (± 0.001)	6.03	3.81	32.2	0.168	75.1	37.7
1400 °C 70 MPa	2.224 (± 0.001)	6.04	3.82	32.3	0.167	75.5	37.8
1400 °C 6 MPa	2.249 (± 0.006)	6.12	3.84	33.1	0.176	77.8	40.0

**Figure 1:**
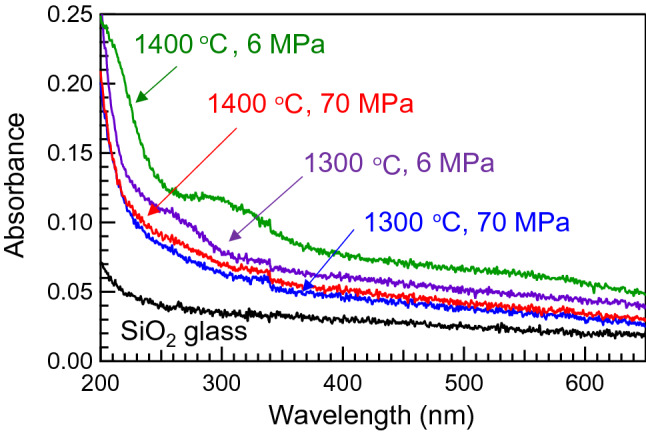
Optical absorption spectra of SPS-SiO_2_ glasses prepared at different conditions along with that of SiO_2_ glass.

Figure 1 shows the optical absorption spectra of the glasses along with that of conventional SiO_2_ glass. Small absorption bands are observed below 350 nm, which originate from the generation of defects, such as oxygen deficiency centres and dangling oxygen bonds^11–13^. It is notable that absorbance of the samples prepared at 6 MPa is higher than that of ones prepared at 70 MPa under the same preparation temperature. We also found that the increasing absorbance, i.e. defect formation, is suppressed by applying lower temperature under the same preparation pressure.

### Positron annihilation spectroscopy (PAS) analysis

**Figure 2:**
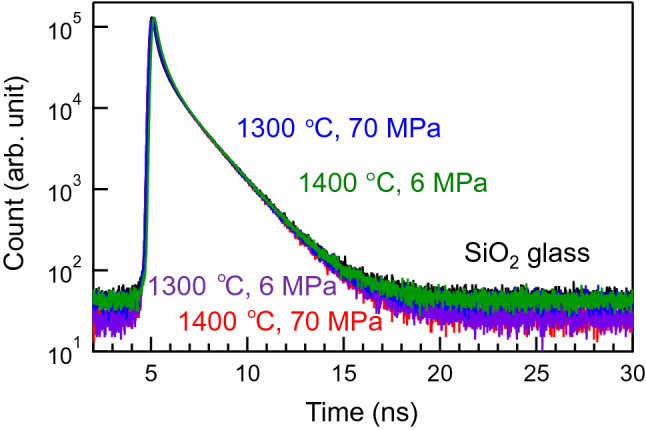
Positron decay curves of SPS-SiO_2_ glasses prepared at different conditions along with that of conventional SiO_2_ glass.

For structural analysis of the SPS-SiO_2_ glasses, we performed PAS, which is used to quantify the cavity size in materials^17–20^. Figure 2 shows the positron decay curves of the SPS-SiO_2_ and conventional SiO_2_ glasses; there is no significant difference in the curves. For insulators, the decay constant of *ortho*-positronium (the third component in fitting the decay curve) is used for size calculation. The cavity radius of the SPS-SiO_2_ glass was calculated to be 0.245 (± 0.002) nm, which is almost identical to that of standard SiO_2_ glass (0.247 nm) (Supplementary Table 1). This result is in accordance with the values published in the literature for densified silica glasses^20^. As reported previously^19^, PAS tends to detect larger cavities (rather than smaller ones) in SiO_2_ glass. Hence, the small changes observed in the density cannot be explained using the cavity sizes obtained by PAS as it is based on preferential annihilation of positrons in cavities. A microscopic analysis is expected to provide additional information to explain the densification of SPS-SiO_2_ glasses.

### Microscope observation

We performed Scanning Electron Microscope (SEM) and Transmission Electron Microscope (TEM) imaging of the surface and the interior to analyse the morphologies of the SPS-SiO_2_ glasses.

**Figure 3:**
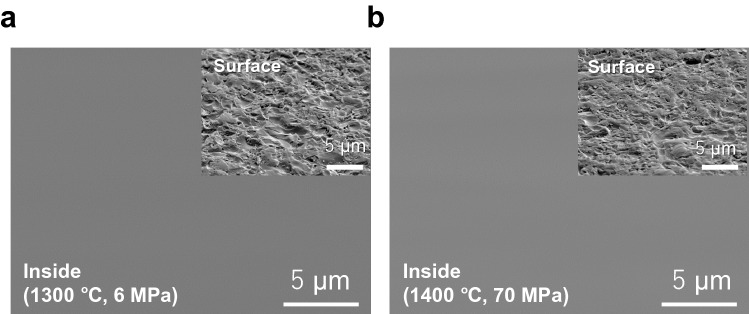
SEM observation of SPS-SiO_2_ glasses. SEM images of interiors of the SPS-SiO_2_ glasses prepared at 6 MPa, 1300 °C (**a**) and 70 MPa, 1400 °C (**b**), respectively. The insets show the surface of the samples. The surface and the interior of the samples were polished mechanically and by ion-milling, respectively.

Figure 3 shows the SEM images of SPS-SiO_2_ glasses prepared at different conditions. Inset shows the surface of the samples by mechanically polishing. Inside the samples prepared by ion-milling, there are no grain boundaries originating from the SiO_2_ powder, resulting in a uniform morphology. The fully sintered images at 1300 and 1400 °C are very similar to those in the previous paper^6^. It is natural that the obtained SPS-SiO_2_ glasses without grain boundary exhibit the transparency over a wide wavelength range.

**Figure 4:**
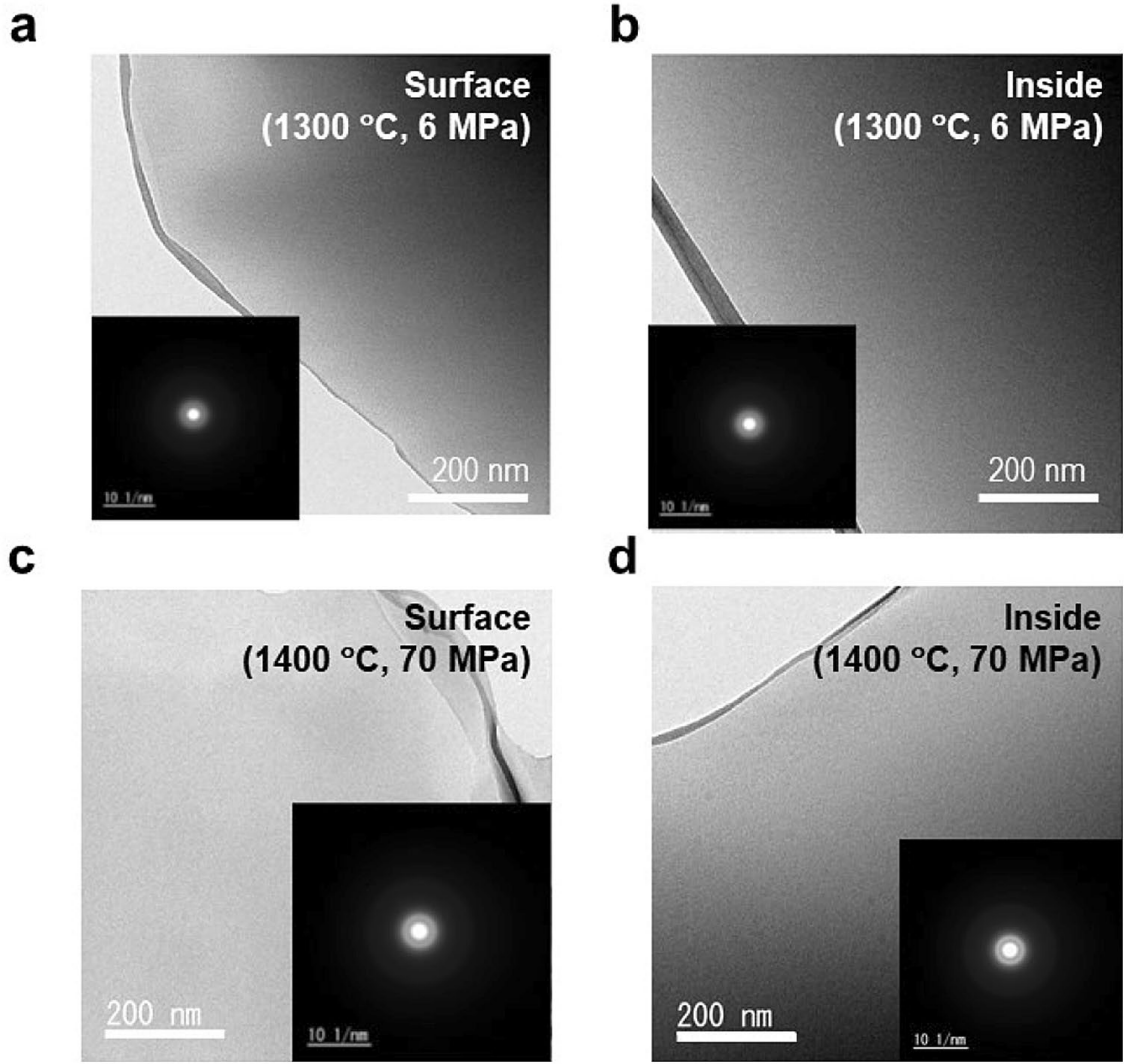
TEM observation of SPS-SiO_2_ glasses. TEM images of the (**a**, **c**) surfaces and (**b**, **d**) insides of the SPS-SiO_2_ glasses prepared at 6 MPa, 1300 °C and 70 MPa, 1400 °C, respectively.

Figure 4 shows the TEM images of SPS-SiO_2_ glasses prepared at different conditions. Both the surface and the interior of the samples are homogenous with no precipitation of crystallites. Hence, all the SPS-SiO_2_ glasses are expected to be amorphous, which was confirmed by conventional XRD (discussed later). Because there is no clear evidence of densification in the TEM results, other analytical approaches are required.

### High energy XRD (HEXRD) analysis

**Figure 5:**
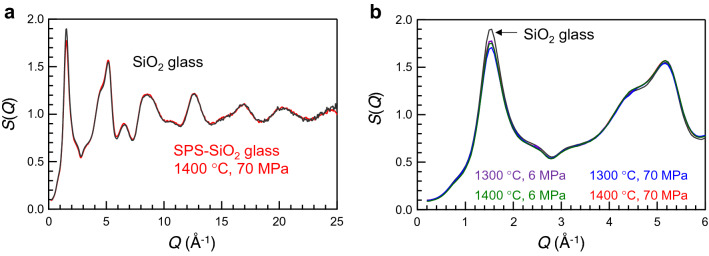
Comparison of *S*(*Q*) of SPS-SiO_2_ glass and SiO_2_ glass. (**a**) *S*(*Q*) of SPS-SiO_2_ glass prepared at 70 MPa, 1400 °C along with that of SiO_2_ glass. (**b**) Enlarged *S*(*Q*) of SPS-SiO_2_ glasses prepared at different conditions along with that of SiO_2_ glass.

Based on the relationship between the first sharp diffraction peak (FSDP) of the X-ray total structure factor *S*(*Q*) and the density of SiO_2_ glasses (as reported in our recent study)^16^, we focused on the FSDP profile of the SPS-SiO_2_ glasses. The FSDP height at *Q* = 1.55 Å^−1^ strongly correlates with the structural disordering of glasses^21–25^. Figure 5a shows the *S*(*Q*) of the SPS-SiO_2_ glasses prepared at 70 MPa, 1400 °C along with that of SiO_2_ glass. The X-rays were irradiated at the centre of the samples. The spectral shapes of both the samples are similar, and no sharp diffraction peak attributable to crystalline SiO_2_ is observed. Similar shapes in the low- to high-*Q* regions confirm that no cluster-like structure is formed in the SPS-SiO_2_ glass. Figure 5b shows the enlarged *S*(*Q*) values of these glasses in the low-*Q* regions. Notably, there is a slight difference in the height of the FSDP. However, the relationships between the FSDP height and the SPS conditions or the density of SPS-SiO_2_ glasses are not completely understood. Although we can conclude that the small difference in the FSDP height originates from the denser packing of the SiO_4_ network by the SPS method, the detailed structure is not clear from these results.

### IR and Raman spectroscopy

**Figure 6:**
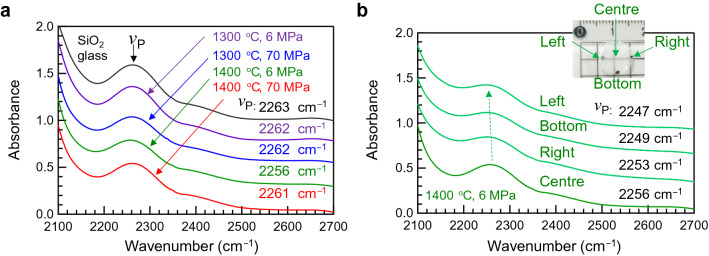
Spatial heterogeneity by micro-IR spectroscopy. (**a**) Micro-IR spectra of SPS-SiO_2_ and SiO_2_ glasses obtained from the centre. (**b**) Enlarged micro-IR spectra obtained from different positions of the SPS-SiO_2_ glass prepared at 6 MPa, 1400 °C. The inset shows the photograph of the SPS-SiO_2_ glass; the black dots at the edges indicate the measurement points.

We used IR and Raman spectroscopy techniques to study the spatial heterogeneity of the SPS-SiO_2_ glass. Figure 6a shows the micro-IR spectra of the SPS-SiO_2_ glasses obtained from the centres of the SPS-SiO_2_ and conventional SiO_2_ glasses. Although the spectral shapes are similar, there is a slight shift in the band at approximately 2260 cm^−1^. This vibration mode is an overtone of the Si–O–Si vibration mode, which is occasionally discussed from the viewpoint of fictive temperature (*T*_f_) of SiO_2_ glasses^15,16,26–33^. Notably, SPS-SiO_2_ glass prepared at 6 MPa, 1400 °C, which exhibits the highest density among all the samples, exhibits the largest shift in the Si–O–Si peak. Figure 6b shows the enlarged microscopic IR spectra obtained from different positions of the SPS-SiO_2_ glass prepared at 6 MPa, 1400 °C. The spectra obtained from the edges of the SPS-SiO_2_ glass are different from that obtained from the centre of the sample; the Si–O–Si peak shifts to lower wavenumbers when moving from the left edge to the right. A shift to a lower wavenumber can be assigned to a higher *T*_f_, i.e., not annealed. However, the peak shift in the present data is too large to explain this phenomenon from the viewpoint of merely the *T*_f_ of SiO_2_ glass^16,29^. In addition, although the SPS-SiO_2_ glasses were obtained by cooling without temperature control, the quenching rate was slower than the water-quenching rate of SiO_2_ glass with a high *T*_f_. Therefore, it is expected that this shift originates from the reaction at the interface between the SiO_2_ powder and the surrounding graphite die.

**Figure 7:**
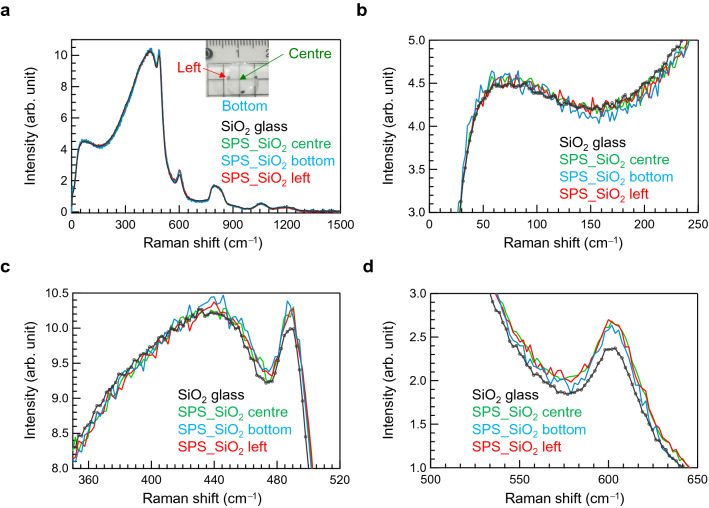
Micro-Raman spectra of SPS-SiO_2_ glass recorded with HH polarisation. (**a**) Micro-Raman spectra of SPS-SiO_2_ glass prepared at 6 MPa, 1400 °C along with that of SiO_2_ glass. The inset shows the photograph of the SPS-SiO_2_ glass marked with the measurement positions. Enlarged Raman spectra showing the (**b**) boson peak, (**c**) D_1_ peak, and (**d**) D_2_ peak.

In Raman spectroscopy, we focused on the boson peak and the vibrational modes at 490 cm^−1^ (D_1_) and at 600 cm^−1^ (D_2_)^34–39^. Therefore, we discuss Raman spectra measured with HH (parallel nicol) polarisation (Supplementary Fig. 1(a)). Although the origins of both these vibrational modes are not completely understood, the boson peak has been correlated with the free volume of glasses^40^. On the other hand, D_1_ and D_2_ are assigned to the vibration of four- and three-membered rings of SiO_4_ tetrahedral units, respectively^39^. In contrast to conventional SiO_2_ glass, SPS-SiO_2_ glass exhibits fluorescence upon laser irradiation, suggesting the formation of defects in the matrix (Supplementary Fig. 1(b)). The expected defect generation is consistent with the results of optical absorption shown in Fig. 1. Considering the results of micro-IR spectroscopy, the Raman spectra were also obtained from three different points (shown in the inset of the photograph) on the samples. The broad baseline due to fluorescence was removed by applying an extended multiplicative signal correction (EMSC) algorithm, adopting the bulk SiO_2_ glass spectrum without fluorescence as a reference. ^41^ Figure 7a shows the micro-Raman spectra of the SPS-SiO_2_ (prepared at 6 MPa, 1400 °C) and conventional SiO_2_ glasses; these spectra were recorded with HH polarisation. Although the spectral shapes are roughly similar, there is a slight difference between the spectra of SPS-SiO_2_ and SiO_2_ glasses. Figures 7b–d show the enlarged Raman spectra highlighting the boson, D_1_, and D_2_ peaks, respectively. The height of the boson peak of SPS-SiO_2_ glass is comparable to that of the SiO_2_ glass, and a remarkable peak shift is not observed. On the contrary, the intensities of the D_1_ peak at 490 cm^−1^ (Fig. 7c) and the three-membered-ring (D_2_) peak at 600 cm^−1^ (Fig. 7d) of SPS-SiO_2_ glass increase. It is suggested that D_2_ structures exist at the vicinity of SiO_2_ surface^39^. Considering the result of PAS, in which a large cavity is not diminished by SPS, it is suggested that small silica units are formed at the edges of the sample (near the interface with the mould). Because the decrease in the height of FSDP in HEXRD (Fig. 5) also suggests a less ordered glass network, it can be concluded that a defect-like structure is generated in the SPS-SiO_2_ glass. Notably, the height of the boson peak is comparable, although the shift in the Si–O–Si vibrational peak is the largest at the left edge of the sample (interface with the mould). The correlation between the Boson peak of silica glass and the average of distribution of Si–O–Si bonding angle is well established^42,43^. Since there is no visible correlation between the boson peak in the Raman spectrum and the Si–O–Si vibrational peak in the IR spectrum, it is expected that Raman spectroscopy is less sensitive to small density changes than IR spectroscopy.

The results of the present study suggest that the interface with the mould affects the properties of ceramics obtained by pressure-assisted fabrication under reduced atmosphere. We assume that the highest density of SPS-SiO_2_ glass prepared at 6 MPa and 1400 °C is mainly due to reduction reactions, rather than conventional densification by applying pressure above several GPa. In addition, it is expected that higher pressure and lower temperature are effective to prevent reduction reaction. Even in the aerodynamic levitation method^44^, where samples are not in contact with any container, the interface between the materials and surrounding atmosphere is considered important. We believe that the influence of the interface on the structure and physical properties should be considered not only in SPS but also other manufacturing methods. Thus, ceramics with core–shell-like structures can be spontaneously prepared by selecting the fabrication method. In addition, we emphasise that different probes are required for different scales of constituents in a solid-state matrix. For example, because the relationship between elastic (macroscopic) properties and spectroscopic (microscopic) approaches is not straightforward, a combination of analytical methods is necessary for complete characterisation^45–47^. A deeper understanding of the structure of the materials is required for more precise control of the properties.

## Conclusion

Transparent SPS-SiO_2_ glasses without grain boundary or precipitation of crystallites were successfully prepared. The results of HEXRD, micro-IR spectroscopy, and micro-Raman spectroscopy suggest the formation of irregular silica species in the SPS-SiO_2_ glass near the interface with the mould. Since the highest density of SPS-SiO_2_ glass was attained prepared at lower pressure and higher temperature, it is expected that that the densification of SiO_2_ glass is mainly due to reduction reactions, rather than conventional densification by applying GPa pressure. Although sintering can produce novel solid-state matrices, spatial analysis and macroscopic properties are important for understanding the nature of the sample.

## Methods

### Preparation

SiO_2_ glasses were prepared by SPS of silica powder with a diameter of approximately 25 μm (99.999% purity; High Purity Chemicals). The SiO_2_ powder was loaded into a graphite die with an inner diameter of 10 mm and sealed with two graphite punches. The sintering temperature was controlled according to the following sequence: the temperature was increased to 600 °C from approximately 25 °C in approximately 5 min, maintained at 600 °C for approximately another 5 min, increased again to 1300 or 1400 °C at a constant rate of 10 °C/min and then maintained for 15 min. The entire sintering process was carried out in vacuum, and a pressure of 6 or 70 MPa was applied between both ends of the graphite punch. After sintering, the sample was cooled without controlling the temperature. Four different preparation conditions were used in this study: 6 MPa, 1300 °C; 6 MPa, 1400 °C; 70 MPa, 1300 °C; and 70 MPa, 1400 °C. The obtained SiO_2_ glass was surface polished and characterised using different methods.

### Characterisation

A high-energy X-ray diffraction (XRD) experiment was performed using a two-axis diffractometer dedicated to the study of disordered materials at the BL04B2 beamline of the SPring-8 synchrotron radiation facility (Hyogo Japan)^48^. The energy of the incident X-rays is 61.43 keV. The raw data were corrected for polarisation, absorption, and background, and the contribution of Compton scattering was subtracted using a standard data analysis software.

The morphology of the samples was measured using a scanning electron microscope (SEM), where SEM images were taken using a JSM-6510(JEOL). An HF-2000 (Hitachi) microscope was used to obtain transmission electron microscopy **(**TEM) images. Transmittance measurements were performed at 25 °C using a spectrophotometer (UH-4150; Hitachi, Ltd.). The ultrasonic velocities of the longitudinal (*V*_L_) and transverse (*V*_T_) waves were measured at room temperature using the ultrasonic pulse-echo method (DPR300, JSR Ultrasonics). The frequencies of the longitudinal and transverse transducers are 10 and 5 MHz, respectively. The Young's modulus *(E*_0_), instantaneous shear modulus (*G*_0_), bulk modulus (*K*_0_), and Poisson’s ratio (*ν*) were calculated according to previously reported methods^47^. The errors in *E*_0_, *G*_0_, and *K*_0_ were less than ± 0.1 GPa.

The micro-Raman spectroscopy was performed in backscattering geometry using a single-frequency diode-pumped solid-state laser operating at 532 nm (Oxxius LCX-532S-300) and a custom-built microscope with ultra-narrowband notch filters (OptiGrate). The incident laser was attenuated to 7 mW and focused using a 50 × objective lens. The scattered light, collected by the same lens, was analysed using a single monochromator (Jovin-Yvon, HR320, 1200 grooves/mm) equipped with a charge-coupled device camera (Andor, DU420). The measurement system for Raman spectra is shown in a previous study^49^. We employed a multivariate analysis software Unscrambler 11 (Camo Analytics), which provides EMSC, to remove fluorescence-like broad baselines for the SPS samples.

Positron annihilation lifetimes were measured using a PSA TypeL-II system (Toyo Seiko Co., Ltd.) with an anti-coincidence system^50^. The ^22^Na source, with a diameter of 15 mm, was encapsulated in a Kapton film. The accumulated count for each sample was 10^7^.

Infrared spectra (in the mid-infrared (IR) region from 600 to 8000 cm^−1^) were measured using the IR beamline BL43IR at the SPring-8 synchrotron facility (Hyogo, Japan). A Fourier transfer infrared (FTIR) microspectrophotometer (BRUKER model HYPERION IR microscope with a VERTEX70 FTIR spectrometer) was used with IR synchrotron radiation. The microscope has a motorised xy-stage, which is used to specify the measurement position. The magnification of the objective mirrors is 36 × . The spatial resolution is approximately 20 µm at 2000–3000 cm^−1^. The wavenumber resolution is 1 cm^−1^, and the number of accumulations is 4000 times. The sample was set on a stainless steel mesh with a honeycomb array with a hole diameter of 2 mm. All the measurements were performed at room temperature. The infrared optical path was purged with dry air to remove water and CO_2_ molecules (FT-IR purge gas generator; Parker Co., Ltd.).

## Supplementary Information

Supplementary Information.

## Data Availability

The authors declare that all relevant data supporting the findings of this study are available from the corresponding authors on request.
