# Supplementary material for: Densification in transparent SiO2 glasses prepared by spark plasma sintering
Source: Sci Rep. 2022 Aug 30;12:14761. doi: 10.1038/s41598-022-18892-4 (PMC9427799; doi:10.1038/s41598-022-18892-4)
Supplement: Supplementary file 1 — Supplementary Information. [file 41598_2022_18892_MOESM1_ESM.docx]

**Supplementary Information**

**Densification in transparent SiO_2_ glasses prepared by spark plasma sintering**

Hirokazu Masai, Hiromi Kimura, Naoyuki Kitamura, Yuka Ikemoto, Shinji Kohara, Atsunobu Masuno, Yasuhiro Fujii, Takamichi Miyazaki, Takayuki Yanagida

**Table S1**

Fitting results of positron lifetime spectra of the present SiO_2_ glasses. The standard errors are shown with parentheses.

| Name | Composition | *τ*_1_ (ns) | *I*_1_ (%) | *τ*_2_ (ns) | *I*_2_ (%) | *τ*_3_ (ns) | *I*_3_ (%) | Cavity radius (nm) |
| --- | --- | --- | --- | --- | --- | --- | --- | --- |
| SiO_2_ glass | reference | 0.139  (±0.002) | 22.9  (±0.3) | 0.454  (±0.005) | 27.1  (±0.2) | 1.617  (±0.002) | 50.0  (±0.1) | 0.247 |
| SPS-SiO_2_  glass | 1300 °C,  6 MPa | 0.152  (±0.002) | 21.8  (±0.3) | 0.471  (±0.005) | 30.6  (±0.3) | 1.611  (±0.002) | 47.6  (±0.1) | 0.247 |
|  | 1400 °C,  6 MPa | 0.143  (±0.002) | 21.1  (±0.3) | 0.460  (±0.004) | 33.2  (±0.2) | 1.609  (±0.002) | 45.7  (±0.1) | 0.247 |
|  | 1300 °C,  70 MPa | 0.142  (±0.002) | 21.1  (±0.3) | 0.452  (±0.004) | 21.4  (±0.3) | 1.576  (±0.002) | 47.5  (±0.1) | 0.243 |
|  | 1400 °C,  70 MPa | 0.151  (±0.002) | 21.9  (±0.3) | 0.467  (±0.004) | 31.7  (±0.3) | 1.596  (±0.002) | 46.4  (±0.1) | 0.245 |

**Figure S1. Raman spectra of SPS-SiO_2_ glass prepared at 6 MPa, 1400 °C.** (a) Micro-Raman spectra of the SPS-SiO_2_ glass recorded with HH (parallel nicol) and HV (crossed nicol) polarisation. (b) Micro-Raman spectra (without baseline correction) of the SPS-SiO_2_ glass recorded with HH polarisation. The increase in the baseline at the bottom and left positions indicates that fluorescence is observed in addition to the signals from the sample.
